# Parental Competences and Stress Levels in Mothers of Children with Autism Spectrum Disorders and Children Developing Neurotypically

**DOI:** 10.3390/jcm13041119

**Published:** 2024-02-16

**Authors:** Beata Tyszkiewicz-Gromisz, Joanna Burdzicka-Wołowik, Piotr Tymosiewicz, Wilhelm Gromisz

**Affiliations:** 1 Department of Fundamentals of Physiotherapy, Faculty of Physical Education and Health in Biała Podlaska, Jozef Pilsudski University of Physical Education in Warsaw, 21-500 Biała Podlaska, Poland; beata.gromisz@awf.edu.pl; 2 Department of Pedagogy and Psychology, Faculty of Physical Education and Health in Biała Podlaska, Jozef Pilsudski University of Physical Education in Warsaw, 21-500 Biała Podlaska, Poland; joanna.wolowik@awf.edu.pl (J.B.-W.); piotr.tymosiewicz@awf.edu.pl (P.T.); 3 Department of Swimming, Faculty of Physical Education and Health in Biała Podlaska, Jozef Pilsudski University of Physical Education in Warsaw, 21-500 Biała Podlaska, Poland

**Keywords:** parental competences, stress, mothers, children, autism

## Abstract

**(1) Background**: the aim of this study was to explore parental competences and stress levels in the mothers of children with autism in relation to the mothers of neurotypical children. **(2) Methods**: the study used the Parental Competence Test and the PSS-10 scale to assess the intensity of stress related to one’s own life situation over the past month. Forty mothers of children with ASD (*n* = 20) and neurotypical children (*n* = 20) participated in the study. **(3) Results**: the mothers of children with ASD showed higher levels of stress (*p* = 0.0002). The mothers of neurotypical children achieved higher scores in parental competences (r = −0.49). The competence of mothers of children with ASD was correlated with rigour (r = 0.50), permissiveness (r = −0.60), overprotectiveness (r = 0.71), and helplessness (r = −0.77). **(4) Conclusions**: mothers of children with autism demonstrate lower parental competences than mothers of neurotypical children. Mothers of children with autism are less rigorous but more permissive, overprotective, and helpless. They tend to become heavily involved with their child. An overprotective attitude and greater tolerance for antisocial behaviours among parents of children with ASDs protect them from excessive stress.

## 1. Introduction

Autism spectrum disorders (ASDs) are the subject of numerous studies. They are of significant importance for science and practice, and for the parents of children with ASDs, aiming to improve the quality of their lives [1,2].

The aetiology of autism is unknown, and researchers are identifying the pathogenic mechanisms of autism [3,4]. Many researchers believe that it results from a multifactorial aetiology [3,5,6]. The primary cause of ASDs is considered to be a psychiatric disorder, with parents playing a major role in the onset of the disorder [7]. ASDs have a familial predisposition, indicating that genetic factors significantly contribute to the development of this condition [8].

Autism spectrum disorders represent a lifelong neurological condition characterised by impaired social and behavioural communication and processing of emotional signals [9]. 

Due to the diversity of the disorder, children with ASDs show a range of abnormalities in behaviour, cognition, and communication [10]. The disorders are more common in boys than girls [11], and are usually coupled with intellectual disabilities [12,13], attention deficits [14], and eating disorders [15].

In particular, mothers of children with autism are aware that caring for their child will last a lifetime. This causes fear, anxiety, and stress about the future and may cause difficulties in their social functioning [16].

Anna Matczak defines social competences as “complex skills determining the effectiveness of coping with certain types of social situations, acquired by an individual in the course of social training”. Parental competences are thus acquired through the skilful performance of parenting activities and participation in the life of the child. We can modify them through the influence of knowledge and experiences from the environment [17]. The upbringing of a child takes place through participation in various social situations, and the effectiveness of functioning is fostered by parental skills, which can develop and change in these social aspects [18]. Parental competences include the affective factor, i.e., the warmth of the relationship, and control in the form of discipline. Additionally, communication is a component of parental competences. The essence of parental competences is to adapt to, and follow, the child’s needs. This requires flexibility on the part of the parent and adaptation to the child’s changing needs and abilities with age. It therefore requires the parent to be constantly engaged in their role. This approach is remarkably consistent with the concept of proximity parenting [19].

When speaking of parental competences, a criterion of effectiveness is introduced, reflected in the adequacy of parental behaviour in terms of the parental goals that they themselves have chosen—a subjective understanding—and also in relation to social expectations, and here, we speak of an objective understanding. It is very often the case that those who act in accordance with the broadly understood good of the child, increasing the child’s ability to cope in society, are considered to be competent parents. Fundamental to the fulfilment of the parental role is the stimulation of the child’s development towards autonomy in thought and action [20]. An authoritative parenting style is considered the most desirable [21]. The parenting style has a tremendous impact on the functioning of children in various areas. It is the foundation for social and emotional development. These areas are compromised in children with ASDs. Therefore, placing greater emphasis on reciprocal parenting, clear rules, and cooperation contributes to better coping in life. In this style, adhering to jointly established principles and rules is important, and such structured behaviour facilitates the functioning of a child with an ASD. On the one hand, it is full of warmth and sensitivity to the child’s needs and, at the same time, demands are made. Consistently demanding the fulfilment of responsibilities while simultaneously expressing acceptance and giving praise promote the child’s development. Consequently, the important dimensions of competence are control–freedom, involvement–distance, and organisation–disorganisation.

The competences of the parent become particularly important when raising a child with a non-standard upbringing. We may observe parents overprotecting their child from responsibilities or social relationships. The underlying behaviour is based on a desire to protect the children, to compensate, e.g., in situations of rejection or to avoid additional disadvantages [22]. Negative attitudes may manifest themselves as inappropriate ways of coping with the child’s disability by, for example, being overly close to and catering for the child, or by being repelled and distant or inconsistent. The source of these attitudes may be the experience of internal conflicts and tensions related to the parents’ personal situation. Stress is thus undoubtedly a concomitant factor in the process of adjusting to a difficult family situation [23]. 

This concept of stress is transactional, as, in a stressful situation, there is a confrontation between the individual’s beliefs, values, and abilities and the demands, constraints, and resources imposed by the situation. In this framework, two types of mediating processes occur between stress and its effects: primary and secondary cognitive appraisal, and coping. The primary appraisal process involves the individual perceiving and interpreting the situation as a harm/loss, threat, or challenge, and each of these assessments is associated with characteristic emotions. If the situation is deemed stressful in this assessment, then it initiates the secondary appraisal process, which concerns the ability to take action to remove the cause of stress or alleviate its effects, as well as to achieve potential benefits. Primary and secondary appraisal are simultaneous and interconnected processes. As for coping with stress, this process has been defined as the constantly changing cognitive and behavioural efforts aimed at mastering external and internal demands, assessed by the individual as burdening or exceeding their resources [24]. This process serves two functions: instrumental (task-orientated, problem-focused) and emotional self-regulation. The instrumental function aims to improve the relationship between demands and the individual’s capabilities, while, through emotional self-regulation, the individual reduces unpleasant tension and alleviates other negative emotional states induced by stress [25,26,27].

The measurement of stress in the study concerned the individual’s response to an event, and thus the parents’ response to factors associated with their child’s autistic disorder. These factors may include a lack of time due to constant care, helping the child with daily activities, problems regarding the provision of an appropriate education for the child, and anxiety about the child’s future. The stress associated with caring for an ill family member drains one of the energy required to function and leads to feelings of depression and anxiety [28,29].

The aim of this study was to explore the parenting competences and stress levels of mothers of children with autism in relation to mothers of neurotypical children.

## 2. Materials and Methods

The study employed a correlational method, using standardised diagnostic tools for variable measurement: PSS-10 for assessing stress levels and TKR—Parental Competence Test. The study involved 40 Polish mothers of children with ASDs and neurotypical children, who were divided into two groups. The study employed purposive sampling. Polish mothers of children with ASDs and mothers of neurotypical children from the Biała Podlaska area were included in the study, having received written invitations from our research team. Forty mothers responded to the invitation to participate in the study and were divided into two groups. The first group consisted of 20 mothers of autistic children attending school at the Wspólny Świat Association in Biała Podlaska, Poland. The other group consisted of 20 mothers of neurotypical children. The group was selected according to the developmental age of the children. 

### 2.1. Parental Competence Test

Mothers’ competences were assessed using the Parental Competence Test (TKR), understood as parents’ dispositions conditioning their use of ways of dealing with the child that are conducive to the child’s development, i.e., the development of the child’s autonomy and self-regulatory skills and sense of efficacy. The tool consists of 30 tasks in the form of short stories describing different child-rearing situations. Most of them are problem situations related to the child’s difficulties, misbehaviour, or difficult or impossible demands, but some stories are about positive events, such as the child’s successes. The stories relate to children of different ages and different areas of activity (such as learning, household chores, peer contact, leisure activities), and the parental behaviours described vary in the degree of control exercised over the child, the rigidity of the demands system, and the emotional involvement. For each situation, three possible ways for the parent to behave are proposed, and the respondent is asked to rate for each of them the likelihood that they would behave in that way; these ratings are conducted on a four-point scale, choosing between the answers definitely no, rather no, rather yes, and definitely yes. In addition to the competence scale, the tool includes four additional scales to assess the parent’s propensity to make mistakes: rigour, permissiveness, overprotectiveness, and helplessness. The Cronbach’s alpha coefficients form the basis for assessing the reliability of the test scales. The most reliable results for the TKR, both in terms of internal consistency and stability, are found in the competence scale, where Cronbach’s alpha is 0.91. The stability of results for the other scales, as well as the internal consistency for the rigour (Cronbach’s alpha = 0.83), permissiveness (Cronbach’s alpha = 0.76), and overprotectiveness (Cronbach’s alpha = 0.79) scales, are entirely satisfactory. However, some doubts arise regarding the homogeneity of the helplessness scale, where the Cronbach’s alpha coefficient is 0.76. The research provided data confirming the accuracy of the TKR [21].

### 2.2. The PSS-10 Questionnaire

The stress level was examined with the PSS-10 questionnaire. It contains 10 questions on various subjective feelings related to difficult personal situations and ways of coping with them. It is used to assess the intensity of stress related to one’s own life situation over the past month. Internal consistency was tested in a study of 120 adults, achieving the Cronbach’s alpha coefficient of 0.86. The correlation of all questions with the scale’s total score is satisfactory. The reliability established on the basis of a double examination of a 30-person group of students at an interval of 2 days was 0.90, and, at an interval of 4 weeks, was 0.72. In our study, we correlated the scale results with the results measured with the COPE scale, Rosenberg’s SES, and Schwarzer’s GSES. The PSS-10 scale accurately measures subjective feelings about personal problems and events and ways of coping with them [30].

### 2.3. Research Organisation

A meeting was held for mothers of students with ASDs at the Wspólny Świat facility. At the meeting with the mothers, the purpose of the study was discussed and consent was sought for the study. The research was carried out by psychologists. Mothers of neurotypical children were recruited for the study in the psychological office prior to the training called ‘Relationships for a medal’. The purpose of the study was presented, and consent to conduct the study was requested. The schedule of the study was announced shortly after the collection of consent from the parents or legal guardians of the students. All mothers were given a specific date for the survey, as well as a separate room to ensure as much comfort as possible and to minimise any disruption in completing the surveys. All activities undertaken during the study were based on the highest ethical standards and were conducted in accordance with the principles of the Declaration of Helsinki [31]. The study was approved by the Scientific Research Ethics Committee of Jozef Pilsudski University of Physical Education in Warsaw (SKE 01-23/2014). The questionnaires were completed by parents of autistic children during classes at the Wspólny Świat Association in Biała Podlaska, Poland.

### 2.4. Statistical Methods

The results of the study were statistically analysed and presented using the arithmetic mean (X), standard deviation (SD), and median (Me). The normality distribution of the variables was examined using the Shapiro–Wilk test. The variables did not have a normal distribution. The non-parametric Kruskal–Wallis test was applied, and an r-Pearson correlation analysis was performed between the study variables. Also, the regression analysis was carried out. Significance levels of *p* < 0.05 and *p* < 0.001 were assumed and the statistical programme R Version 4.5.0. [32] was used for statistical analysis.

## 3. Results

Forty Polish mothers (twenty mothers of autistic children—mean age 42.1 ± 4.02, and twenty mothers of neurotypically developing children—mean age 36.9 ± 2.73) were assessed using the Parental Competence Test (TKR) and PSS-10 questionnaire. More detailed characteristics of the groups under study are presented in Table 1.

The stress levels of mothers of children with autism spectrum disorders (ASDs) were significantly different from those of mothers of neurotypical children (*p* < 0.001). Mothers with a child on the spectrum showed higher levels of stress related to their own living situation over the past month than mothers of neurotypical children. 

Mothers of children with neurotypical development had higher parental competences. Mothers of children with autism had lower parental competences, which differed significantly (*p* < 0.05). Mothers of neurotypical children manifested higher rigour, understood as a tendency to be overly controlling combined with low involvement. Those with high rigour were characterised by rigidity and emotional distance. The differences were significant in relation to mothers of children with autism (*p* < 0.001). Surprisingly, mothers of children with autism showed greater permissiveness in relation to mothers of neurotypical children. The differences were significant at (*p* < 0.05). Permissiveness is understood as granting excessive freedom to the child. Typically, this trait correlates negatively with overprotectiveness. Among mothers of children with ASDs, the results are different. They had higher levels of overprotectiveness (*p* < 0.05) and helplessness (*p* < 0.001). The differences were significant compared to the mothers of neurotypical children (Table 2). Thus, we can say that mothers of children with ASDs tend to be strongly involved in their child’s life. They are much more likely to take care of them, show an attitude of care and help, and show more concern for the child than mothers in the other group. Parents scoring high in helplessness have low problem-solving skills and are characterised by low flexibility (they fall into patterns, rigid habits, and low reflectivity) [20]. 

Among mothers of children with autism, two variables significantly correlated with stress—parental competences (r = 0.48, *p* = 0.001) and helplessness (r = −0.45, *p* = 0.03)—while, in the mothers of neurotypical children, stress only correlated with competences (r = −0.49, *p* = 0.02). We can therefore say that the more stressed the mothers of children with autism feel, the more competent and the less helpless they feel. Optimal stress levels constructively influence their performance and facilitate rational thinking. Mothers of neurotypical children describe themselves as less competent as their stress levels increase. The competence of mothers of children with ASDs was correlated with rigour (r = 0.50, *p* = 0.02), permissiveness (r = −0.60, *p* = 0.01), overprotectiveness (r = 0.71, *p* = 0.0001), and helplessness (r = −0.77, *p* = 0.002). The more competent the mothers of children with ASDs felt, the more rigorous and the less permissive they were. In mothers of neurotypical children, rigour was not correlated with any indicator, whereas in mothers of children with autism, it was correlated with permissiveness (r = −0.56, *p* = 0.03), overprotectiveness (r = 0.38, *p* = 0.03), and helplessness (r = −0.40, *p* = 0.03). The more rigorous the mothers of neurotypical children were, the less permissive and helpless and the more overprotective they were. Among mothers of neurotypical children, permissiveness correlated with helplessness (r = 0.67, *p* = 0.001). The higher the level of over-permissiveness, the higher the helplessness of the mothers. In both groups, overprotectiveness was correlated with helplessness. In mothers of children with ASDs (r = −0,67, *p* = 0.0006), as overprotectiveness increases, feelings of helplessness decrease. In contrast, in mothers of neurotypical children (r = 0.54, *p* = 0.01), with an increase in overprotectiveness, helplessness also increases.

The regression analysis is presented in Table 3 and Table 4. The linear regression model was statistically significant (*p* < 0.001), with a coefficient of determination R^2^ = 0.80 (Table 3). The reduction of non-significant coefficients was performed, resulting in a statistically significant reduced regression model (*p* < 0.001) with a coefficient of determination R^2^ = 0.81 (Table 4).

Table 4 presents the regression model, indicating that as they possess higher parenting competences, mothers of children with ASDs experience higher levels of stress. The regression model shows that the higher the permissiveness of mothers of children with ASDs, the higher the stress. Additionally, the level of overprotectiveness is inversely related, with higher levels resulting in lower stress for the mothers of children with ASDs. As for the mothers of neurotypical children, the regression model was not significant.

## 4. Discussion

In the present study, it was assumed that mothers of children with autism have higher levels of stress than the mothers of neurotypical children. The analysis of the results confirmed this assumption [33,34,35]. The women surveyed who have a child with an ASD show higher levels of stress related to their own life situation over the past month than the mothers of neurotypical children. The fulfilment of the role of a mother to a child with a disability is a fundamental need for many, and it is not an easy task. Similar conclusions were reached by Dunn, Burbine, Bowers, and Tantleff-Dunn, showing that parents of children with autism experience more stress and are more likely to experience negative consequences than parents of children with other disabilities [36]. Children’s developmental disorders are an important determinant of both the physical and mental health of carers. The literature shows higher levels of stress in mothers of children with autism than in mothers of children with other developmental disorders [37,38]. It is important to remember that the parents of children with autism are exposed to chronic stress caused by a lack of social understanding, tolerance, and acceptance of their child’s behaviours. Parental stress can be defined as a complex of immense burdens felt by parents in relation to the role they play [39,40]. In such a situation, the support of a spouse would be very helpful, but the research carried out and the experiences of the mothers interviewed show that in most situations, women are left alone, and this intensifies the stress felt. However, this is not the rule. There are also situations in which family ties are strengthened, the social network is expanded, and family members experience spiritual development [41]. 

The parents of autistic children scored lower on the Parental Competence Test. A low assessment of their own parental competences is one of the three symptoms of parental burnout. This involves discrediting one’s own qualities, leading to increased frustration related to their life situation [33]. However, this does not mean that they are incompetent; rather, they exhibit a less strict and inconsistent attitude, thereby avoiding the child’s challenging behaviours. In this way, parents adapt to difficult situations, adjusting their interactions to the child’s needs. The results of Hickey et al.’s [42] study demonstrated the impact of parental attitudes on behavioural problems in children. The choice of preferred parenting attitude (as discussed by Gagat-Matuła et al. [43]) is dependent on the communication and language skills of the child with an ASD. 

Due to difficulties in communication and conforming to social rules, caregivers of children with autism are more likely to let go of the enforcement of obedience, opting for meekness and a lack of confrontation. The research on parental attitudes indicates numerous positive changes in parents as a result of having a child with autism. They become more tolerant, empathetic, patient, and understanding, according to Szmania [41]. Their lives have been re-evaluated [41]. Winczura [44] puts it differently, stating that the behaviour of parents of children with autism is associated with the more frequent giving of commands, controlling children, and also creating submissiveness in children. Even if they initiate interaction with the child, at the same time, they formulate more orders and control the child’s behaviour more often [44].

The results of the study also showed that mothers of children with autism are more overprotective and helpless. Thus, we can say that mothers of children with ASDs tend to become heavily involved in their child’s affairs. They are much more likely to take care of them, to show an attitude of care and help and to be more anxious about their child than mothers in the other group. The literature on the subject points to the tendency of overprotective parents to give in to the child and abandon the ultimatums set. This attitude also results from the high scores obtained on the helplessness scale, indicating a lack of skill in enforcing demands and a tendency to be subservient to the child. The regression model indicates an increase in the level of stress among parents of children with ASDs, coupled with a higher awareness of parental competences. Simultaneously, the more permissive their attitude towards the children and the more they tolerate antisocial behaviours, the higher the level of stress. This could be due to an awareness of the difficulties in meeting the child’s needs and challenges in communication. Overprotective behaviour allows parents to reduce perceived tension (the greater the overprotectiveness, the lower the stress), making such an attitude adaptive. Due to the small size of the groups, the results should be generalised with caution; nevertheless, they may provide guidance for psychologists, educators, and therapists professionally supporting the mothers of children with autism [45]. An additional limitation in the study was the absence of fathers due to death, divorce, or extended business trips away from the place of residence. 

## 5. Conclusions

1. Women surveyed who have a child with an ASD show higher levels of stress related to their own living situation.
2. Parents of children with autism are exposed to chronic stress.
3. Mothers of children with autism demonstrate lower parental competences than mothers of neurotypical children. Mothers of children with autism are less rigorous but more permissive.
4. Mothers of children with autism are more overprotective and helpless. They tend to become heavily involved with their child.
5. An overprotective attitude and a greater tolerance for antisocial behaviours among parents of children with ASDs protect them from excessive stress.
6. High helplessness indicates the lack of skill in enforcing demands and a tendency to be subservient to the child with an ASD.

## Figures and Tables

**Table 1:** Characteristics of the groups under study.

Parameter	Mothers of Children with ASDs	Mothers of Neurotypical Children
Age (years)	42.1 ± 4.02	36.9 ± 2.73
Married (%)	65	70
Divorced (%)	35	30
Primary school education (%)	0	0
Vocational school education (%)	25	0
Secondary school education (%)	40	0
Higer education (undergraduate) (%)	10	0
Higher education (postgraduate) (%)	25	100
Place of residence—rural area (%)	50	0
Town below 10,000 residents (%)	30	0
Town of 10,000 to 49,000 (%)	20	0
Town of 50,000 to 99,000 (%)	0	100
City over 100,000 (%)	0	0
Parameter	Children with ASDs	Neurotypical children
Age of child (years)	12.4 ± 1.39	12.05 ± 1.54
Girls (%)	20	55
Boys (%)	80	45

**Table 2:** Results of the PSS-10 Test and the Parental Competence Test in the group of mothers of children with autism and the group of mothers of neurotypical children.

Test	Parameter under Study	Study	Arithmetic Mean	Standard Deviation	Median	Kruskal–Wallis Test
PSS-10	Stress level	ASD children’s mothers	6.00	1.78	6.00	*p* = 0.0002
		Neurotypical children’s mothers	4.10	1.17	4.00	
Parental Competence Test	Parental Competences	ASD children’s mothers	6.15	1.57	6.00	*p* = 0.04
		Neurotypical children’s mothers	7.00	1.95	7.50	
	Rigour	ASD children’s mothers	4.45	1.47	4.00	*p* = 0.001
		Neurotypical children’s mothers	6.90	1.33	7.00	
	Permissiveness	ASD children’s mothers	6.70	1.26	7.00	*p* = 0.02
		Neurotypical children’s mothers	5.00	2.43	4.50	
	Overprotectiveness	ASD children’s mothers	6.25	2.55	5.00	*p* = 0.005
		Neurotypical children’s mothers	4.00	2.20	4.00	
	Helplessness	ASD children’s mothers	7.25	1.71	7.00	*p* = 0.001
		Neurotypical children’s mothers	4.10	3.09	3.00	

**Table 3:** Results of the regression analysis of the investigated parameters of mothers of children with autism related to stress.

Parameter under Study	Estimate Std.	Error	*p*
Parental Competences	1.587	0.213	0.001
Rigour	0.174	0.163	0.302
Permissiveness	1.077	0.205	0.0001
Overprotectiveness	−0.309	0.121	0.023
Helplessness	0.074	0.175	0.681

**Table 4:** Results of the regression analysis of the investigated parameters of mothers of children with autism related to stress after reducing non-significant coefficients.

Parameter under Study	Estimate Std.	Error	*p*
Parental Competences	1.578	0.191	0.001
Permissiveness	0.984	0.170	0.001
Overprotectiveness	−0.309	0.106	0.010

## Data Availability

Data are available upon reasonable request.
